# Physiological Response of Atlantic Salmon (*Salmo salar*) to Long-Term Exposure to an Anesthetic Obtained from *Heterosigma akashiwo*

**DOI:** 10.3390/toxins14080575

**Published:** 2022-08-22

**Authors:** Ana Teresa Gonçalves, Alejandra Llanos-Rivera, Miguel Ruano, Veronica Avello, Juan José Gallardo-Rodriguez, Allisson Astuya-Villalón

**Affiliations:** 1 Interdisciplinary Center for Aquaculture Research, O’Higgins 1695, Concepción 4030000, Chile; 2 GreenCoLab—Associação Oceano Verde, Campus de Gambelas, Universidade do Algarve, 8005-139 Faro, Portugal; 3 Laboratorio de Biotoxinas de la Universidad de Concepción (LBTx-UdeC), Department of Oceanography, Faculty of Natural and Oceanographic Sciences, Universidad de Concepción, Barrio Universitario s/n, Concepción 4030000, Chile; 4 Fishsource Units and Science Division M&E, Sustainable Fisheries Partnership, Honolulu, HI 96816, USA; 5 Departamento de Ingeniería Química, Escuela Superior de Ingeniería, Universidad de Almería, Carretera Sacramento, Calle San Urbano s/n, La Cañada, 04120 Almería, Spain; 6 Centro de Investigación Oceanográfica COPAS COASTAL, Universidad de Concepción, Concepción 4030000, Chile

**Keywords:** natural anesthetic, welfare practices, Atlantic salmon (*Salmo salar*), physiological response

## Abstract

Despite the invaluable role of anesthetics as a tool for ensuring animal welfare in stressful situations, there is currently a lack of anesthetic drugs that meet the requirements of intensive aquaculture. In response to the growing interest in anesthetic substances of natural origin, this study evaluated the physiological and health impact of an anesthetic based on an extract of the microalga *Heterosigma akashiwo* on juvenile salmon (*Salmo salar*) exposed for a period of 72 h. To simulate a condition closer to reality where fish are subjected to stimuli (e.g., transport), the animals were exposed to 50 mg L^−1^ of algal extract and to physical stress. Functional, physiological, and histological parameters were evaluated in blood and tissues at different sampling periods (0, 24, and 72 h). There was no mortality and the induction and recovery times observed were within the established criteria for anesthetic efficacy. The anesthetic extract did not induce any side effects, such as stress or metabolic damage, indicating that this extract is a viable option for supporting fish welfare during deleterious events. This study provides information to support that the anesthetic extract tested, derived from *H. akashiwo*, is a promising candidate drug for operations requiring sedation (e.g., Salmonid transport).

## 1. Introduction

In the fish production industry, several technical procedures require handling of the fish (e.g., sampling, vaccination, surgery, transport, harvest and slaughter); these are perceived as stressors which jeopardize fish homeostasis [1]. These frequent stressors have a strong effect on growth, immunocompetence, and overall robustness, and consequently on final product quality; they may even lead to mortality in extreme cases [2,3]. The use of anesthetics during such procedures to ensure the welfare of the fish is common practice; however, the final choice of any product, sedative or anesthetic must consider legislation, availability, cost-effectiveness, ease of use and safety for both the operator and the environment, among other factors [4,5,6,7]. Moreover, animal welfare management has become a priority in aquaculture in recent years; therefore, reducing stress during fish-handling throughout the production cycle is a key factor to be considered for a more sustainable industry. In addition, welfare maintenance during stress-inducing processes will have an impact on product quality (e.g., fish appearance and fillet quality) with important consequences for product value and producers’ revenue [8].

Fish anesthetics can be divided into the categories of synthetic and natural (i.e., plant-based) products. Among the most-used synthetic compounds are tricaine, tricaine methanesulfanate (MS-222), quinaldine sulfate, benzocaine (BZ-20) and 2-phenoxyethanol [9]. However, the use of these products has been restrained for several reasons, such as specific national legislations or their limited effects in some species. This has motivated the search for new anesthetics derived from natural sources, the most studied of which are plant extracts or essential oils. The main target plants have been aromatic plants such as *Aloysia*, *Ocimum*, *Lippia*, *Pelargonium*, *Coriandrum*, *Bursera*, *Lavandula*, *Origanum*, *Hesperozygis* and *Syzygium* or *Eugenia*, among others (this has been comprehensively reviewed by Aydin and Barbas [10]). The pioneer species marketed in commercial form was the clove (*Syzygium aromaticum*), presently identified as AQUI-S^®^.

Despite the high value of anesthetics as a tool for ensuring animal welfare under stressful events, many anesthetic drugs elicit stress responses [11]. Comparative studies of several commercial anesthetics available in the market showed medium and long term adverse physiological and/or behavioral effects [4,12,13,14]. The response triggered is a typical stress response, where in a first stage the neuroendocrine system is stimulated to release catecholamines and corticosteroids (e.g., cortisol) into the blood stream. These hormones act on tissue cells, modulating their metabolism and promoting alterations in the organism to provide energy to cope with stress. In a more advanced stage, the immune response might become compromised, and overall homeostatic disruption occurs, reducing the probability of survival [1,15]

These responses to anesthetics are typically measured in the blood, nevertheless the effects can vary greatly and may be species-specific, depending on the anesthetics [16]. For natural substances (typically complex extracts), however, these effects tend to be subtler. AQUI-S and sage essential oil have no adverse effects in marine fish species; they do not induce an increase in plasma cortisol, and even inhibit its release in some cases [17,18,19]. *Lippia alba* and *Hesperozygis ringens* essential oils likewise have no effect on the activity of enzymes related with the hydro-electrolytic balance and have been acknowledged as acceptable anesthetics for fish [20]. Similarly, myrcene, a terpene present in several herbal oils, has been shown to not induce deleterious secondary effects in rainbow trout tissues (*Oncorhynchus mykiss*) even when used for long periods [21]. These results agree with those reported by Souza et al. (2019) [22], that the recurrent secondary effects of natural anesthetics are lower than those of synthetic anesthetics. Nevertheless, the potency or duration of anesthesia induced by these natural extracts may be lower than those of synthetic compounds in some cases [23]; their natural availability is limited and production is costly, reducing their applicability in the immediate future. On the other hand, natural anesthetics and sedatives represent less threat from hazardous residues, and are an alternative when considering quality assurance for consumers. Here, we propose the evaluation of a new alga-derived anesthetic, obtained from extracts of the unicellular alga *H. akashiwo*.

This species is an ichthyotoxic raphidophyceaen microalga, commonly associated with harmful algal blooms (HABs). Fish mortalities caused by production of neurotoxins and/or induction of reactive oxygen species by *H. akashiwo* have been reported worldwide [24,25,26,27,28,29,30]. Some studies have claimed that *H. akashiwo* produces bioactive molecules that might be related to alterations in the voltage-dependent sodium channels [31]. Although initially thought to be an effect due to the production of brevetoxins-like compounds, Gallardo-Rodriguez et al. [32] showed this was not the case for *H. akashiwo*. This would explain the low toxicity found on these extracts, as assessed previously in vitro (Neuro-2a cell-based assay) and in vivo (Zebra fish model) under laboratory conditions [32].

To respond to concerns for animal welfare in aquaculture, it is important to search for efficient anesthetic substances of natural origin that have a shorter safety period for product processing and cause minimal environmental impact when released into water sources. An anesthetic based on the methanolic extract obtained from *H. akashiwo* biomass is a natural product with high potential use in aquaculture, and mass production on an industrial scale should be explored. The action on the voltage-dependent sodium channels [31] is similar to the mode of action of tricaine (MS-222), the anesthetic most widely used in fish to date. Therefore, as the first stage in the assessment of a new marine-derived natural anesthetic, the aim of this study was to assess the physiological and health impact of an extract of the microalga *H. akashiwo* in juvenile salmon (*S. salar*) in simulated industrial conditions.

## 2. Results

### 2.1. Physiological Analysis and Stress Response

All fish used in the experiments were previously screened for pathologies and did not present clinical signs of any disease or health condition. There was no mortality during the experiments. We observed a behavioral response very similar to that expected for a comparable sedative/anesthetic product when the juvenile salmon were exposed to the *H. akashiwo* extract at 50 mg L^−1^. The fish showed deep sedation after 4 min, with loss of reactivity to external stimuli except when strong pressure was exerted. Fish maintained normal equilibrium, but a slight decrease in opercular rate was observed. To simulate the conditions closer to a possible real farming event (e.g., transportation) fish were not fed throughout the trial and the water was kept under closed recirculation conditions. The *H. akashiwo* extract was introduced into the system at the beginning of the trial and fish remained in the same solution for the whole period. Water quality parameters were controlled (i.e., dissolved oxygen, pH, nitrites, nitrates) and kept within the normal range.

**Figure 1:**
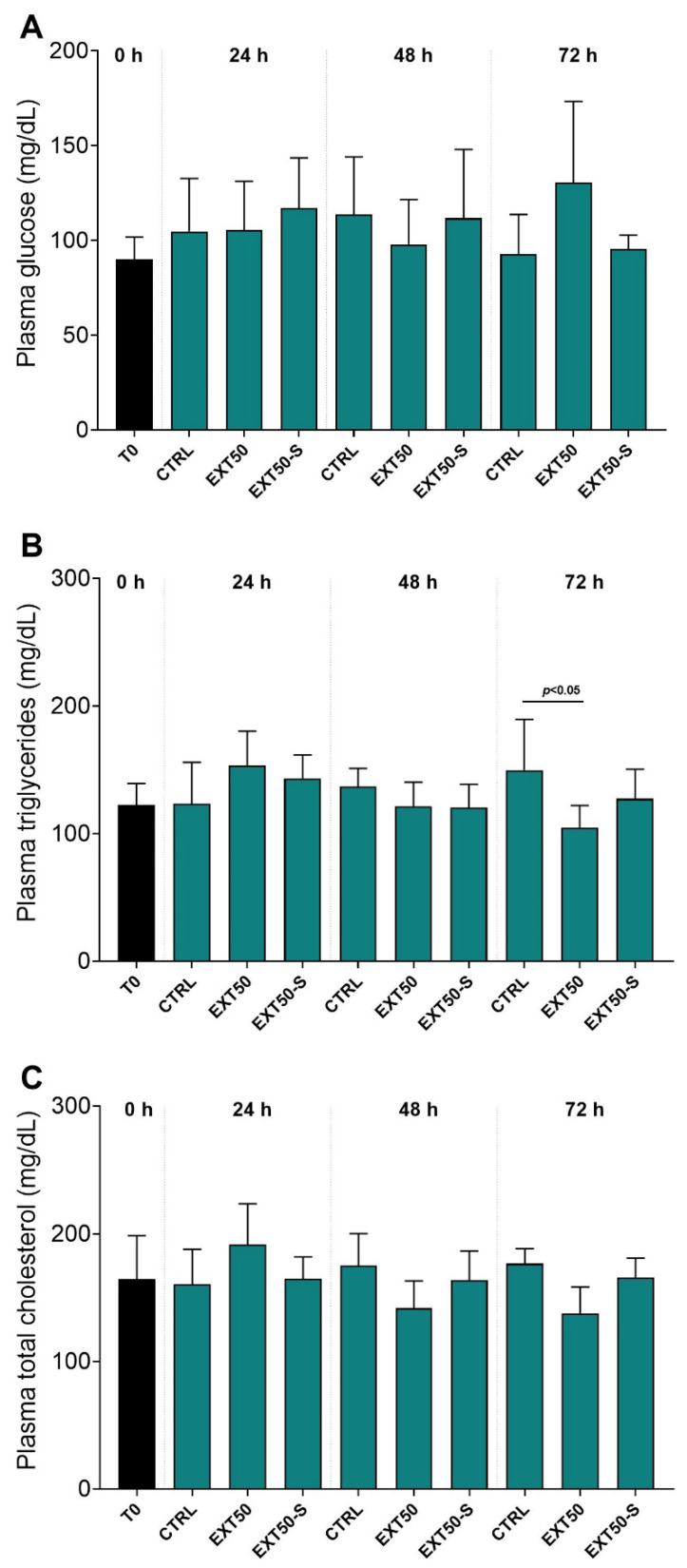
(**A**) Plasma glucose; (**B**) Triglycerides, and (**C**) Total cholesterol levels (mg dL^−1^) of juvenile Atlantic salmon before (0 h) and during 24 h, 48 h and 72 h exposure to the anesthetic (50 mg L^−1^) without stress (EXT50), or with handling stress (EXT50-S). Bars indicate mean ± SD (*n* = 6). Lines indicate differences between the groups under 2-way ANOVA and Tukey’s HSD test (*p* < 0.05).

Fish plasma glucose, triglycerides and total cholesterol levels are shown in Figure 1. Plasma glucose and total cholesterol levels did not alter in fish exposed to the anesthetic formulation, in both the stressed and non-stressed groups. Plasma triglyceride levels were also similar between groups at 24 h and 48 h. However, at 72 h, the CTRL group presented higher levels than EXT50 (*p* < 0.05).

**Figure 2:**
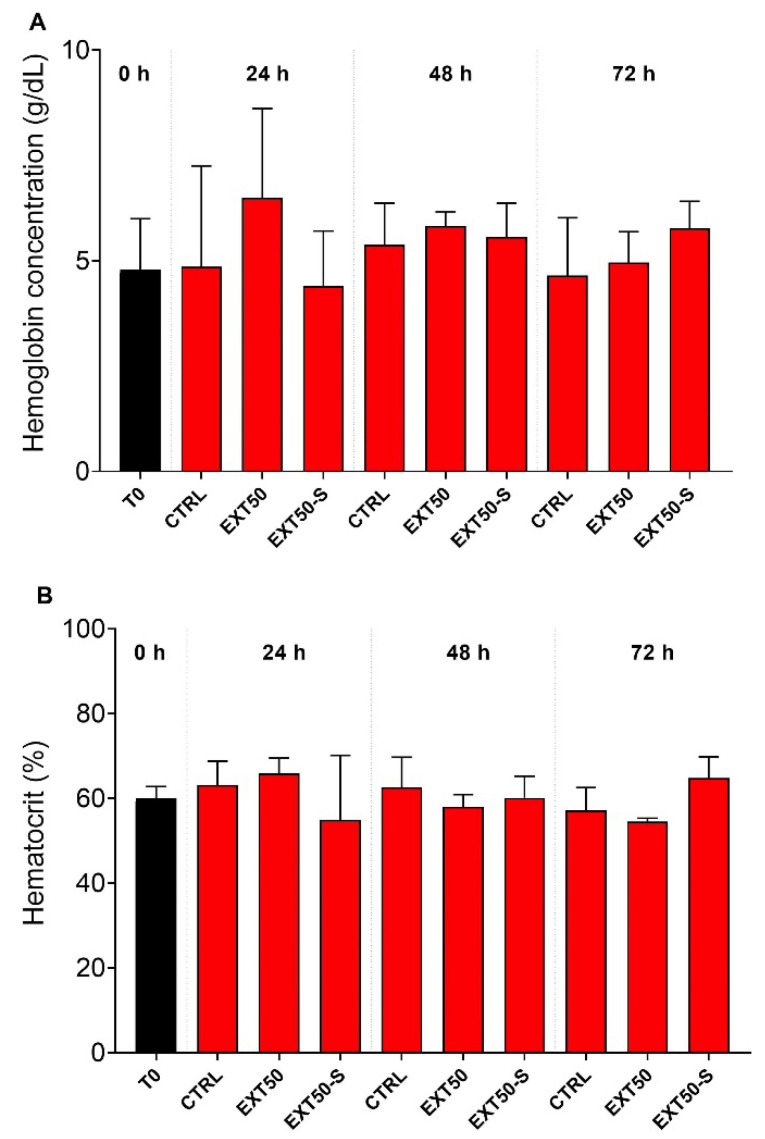
(**A**) Blood hemoglobin concentration (g dL^−1^) and (**B**) hematocrit (%) of juvenile Atlantic salmon before (0 h) and during 24 h, 48 h and 72 h exposure to the anesthetic (50 mg L^−1^) without stress (EXT50), or with handling stress (EXT50-S). Bars indicate mean ± SD (*n* = 6). No significant differences were observed between groups under 2-way ANOVA (*p* > 0.05).

Hemoglobin concentration and hematocrit in blood were not affected by exposure to the anesthetic or to the combination of the anesthetic extract with stress (Figure 2).

**Figure 3:**
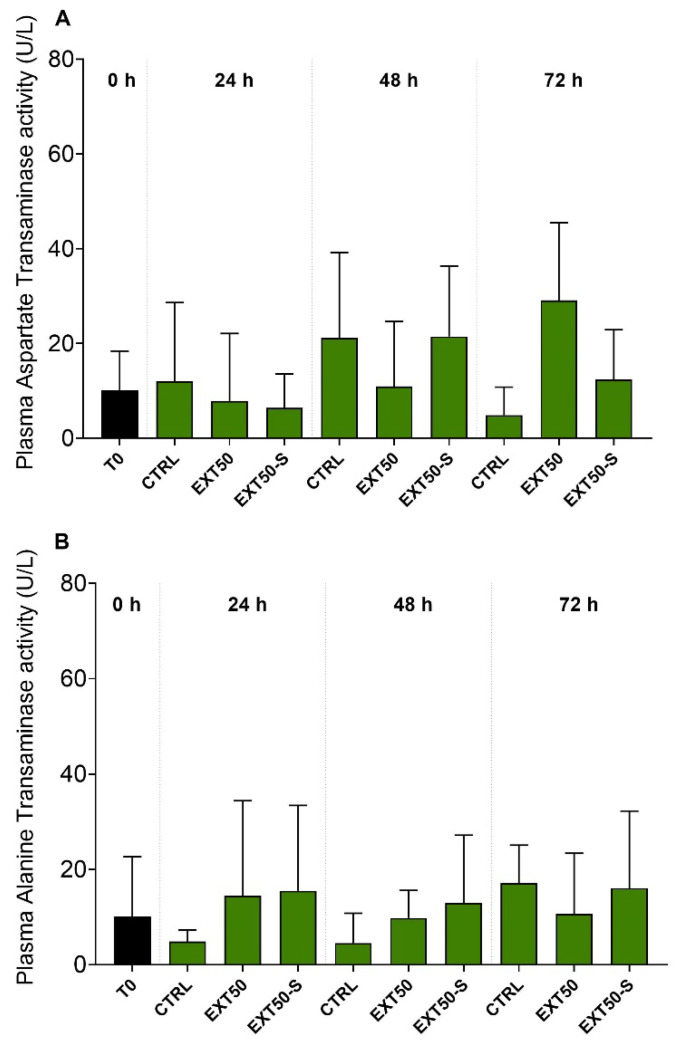
Plasma aspartate (**A**) and alanine (**B**) transaminase activities (U L^−1^) in juvenile Atlantic salmon before (0 h) and during 24 h, 48 h and 72 h exposure to the anesthetic (50 mg L^−1^) without stress (EXT50), or with handling stress (EXT50-S). Bars indicate mean ± SD (*n* = 6). No significant differences were observed between groups under 2-way ANOVA (*p* > 0.05).

In addition, plasma alanine and aspartate transaminase activity (Figure 3) were also similar among groups, indicating no effect of prolonged exposure to the anesthetic and to stress.

**Figure 4:**
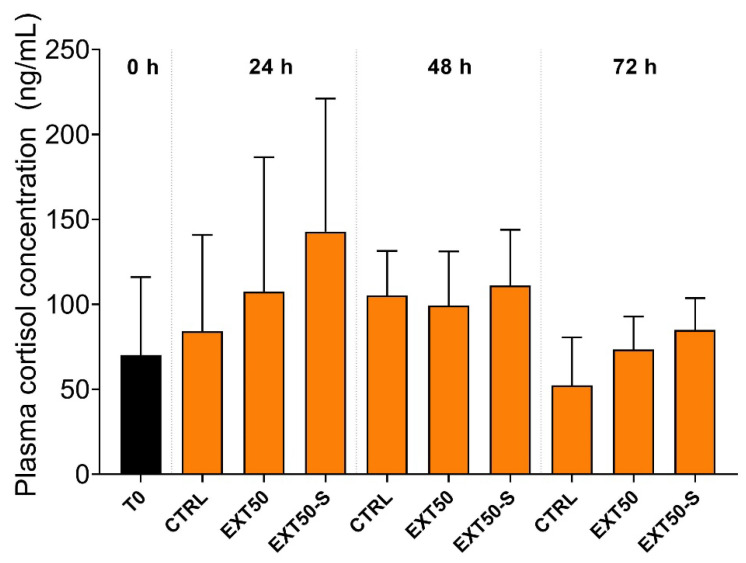
Plasma cortisol levels (ng mL^−1^) in juvenile Atlantic salmon before (0 h) and during 24 h, 48 h and 72 h exposure to the anesthetic (50 mg L^−1^) without stress (EXT50), or with handling stress (EXT50-S). Bars indicate mean ± SD (*n* = 3). No significant differences were observed between groups under 2-way ANOVA (*p* > 0.05).

Plasma cortisol levels also did not differ significantly between groups (Figure 4), despite a visibly steady increase in the EXT50-S group at 24 h. However, at 48 h and 72 h, all groups presented levels similar to the initial moment (0 h, before exposure).

### 2.2. Histological Assessment

**Figure 5:**
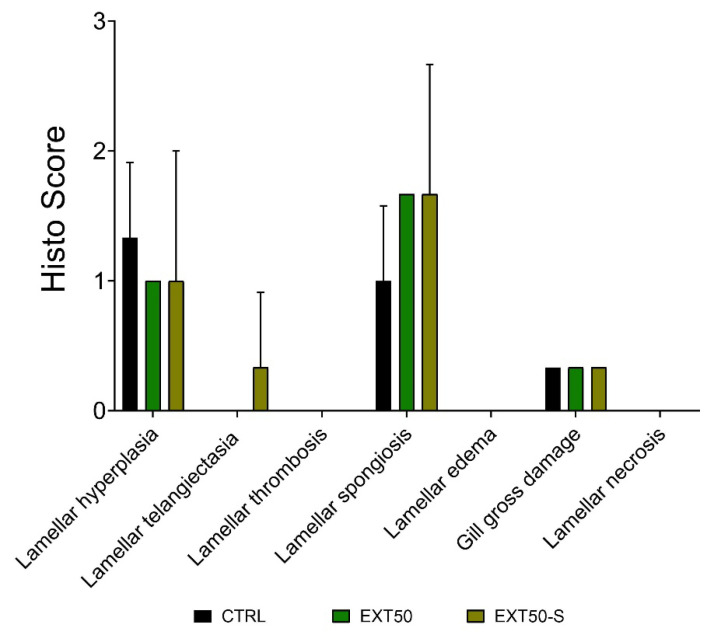
Histological score representing the histological changes occurring in the gills of juvenile Atlantic salmon after 72 h exposure to the anesthetic (50 mg L^−1^) without stress, or with handling stress (+S). Bars indicate mean ± SD of the difference in scores compared with the initial condition before exposure (0 h) (*n* = 3). No bars indicate score 0 (i.e., no histological changes), and no error lines indicate same score in all measured individuals. No significant differences were observed between groups (Kruskal-Wallis, *p* > 0.05).

**Figure 6:**
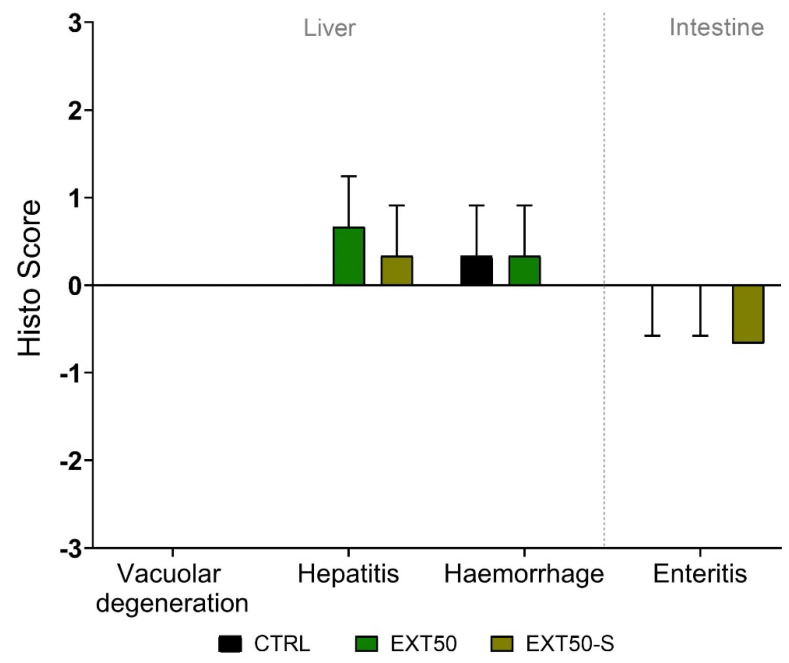
Histological score representing histological changes to the liver and intestine in juvenile Atlantic salmon after 72 h exposure to the anesthetic (50 mg L^−1^) without stress, or with handling stress (+S). Bars indicate mean ± SD of the difference in scores compared with the initial condition before exposure (0 h) (*n* = 3). No bars indicate score 0 (i.e., no histological changes), and no error lines indicate same score in all measured individuals. No significant differences were observed between groups (Kruskal-Wallis, *p* > 0.05).

Histological scores in Atlantic salmon gills before and after 72 h exposure to the anesthetic extract did not differ significantly between groups, indicating that neither exposure to the extract nor to stress produced damage in the gill tissue (Figure 5). Fish in all groups presented lamellar hyperplasia, lamellar spongiosis, and to a lesser degree (lower score) general gross histological alterations of the gills (e.g., hyperplasia, lamellar fusion). These were not altered by exposure to the anesthetic nor to stress. The same was observed in the liver and intestines, as there were no differences in the histological score between groups (Figure 6).

### 2.3. Assessment of Oxidative Stress Markers

**Figure 7:**
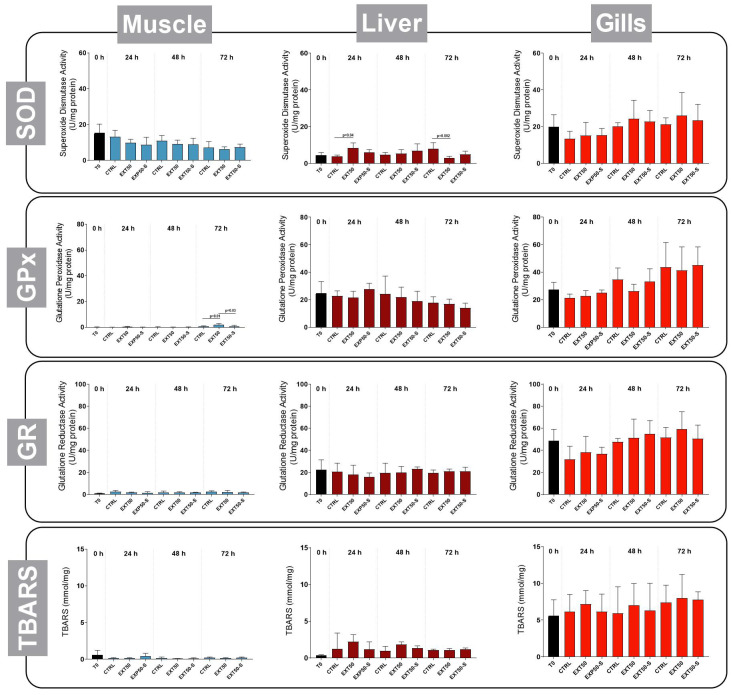
Activity of the enzymes responsive to oxidative stress superoxide dismutase (SOD), glutathione peroxidase (GPx), glutathione reductase (GR) and the concentration of tiobarbituric acid reactive species (TBARS) in muscle, liver, and gills of juvenile Atlantic salmon before (0 h) and during 24 h, 48 h and 72 h exposure to the anesthetic extract (50 mg L^−1^) without stress (EXT50), or with handling stress (EXT50-S). Bars indicate mean ± SD (*n* = 6). Lines indicate differences between groups under 2-way ANOVA, Tukey’s HSD test (*p* < 0.05).

The exposure of Atlantic salmon juveniles to the anesthetic extract for 72 h produced no effects on the activity of SOD in muscle or liver, however at 24 h the activity of this enzyme in liver was higher in the EXT50 group than the CTRL, whereas at 72 h it was lower than in the CTRL group (Figure 7). The activity of GPx was higher in the muscle of the EXT50 group fish after 72 h, however in liver and gills there was no difference. Similarly, the activity of GR in tissues was not altered by continuous exposure to the anesthetic, even when the fish were physically stressed (Figure 7). In addition, there were no differences in the TBARS levels in any tissue.

**Figure 8:**
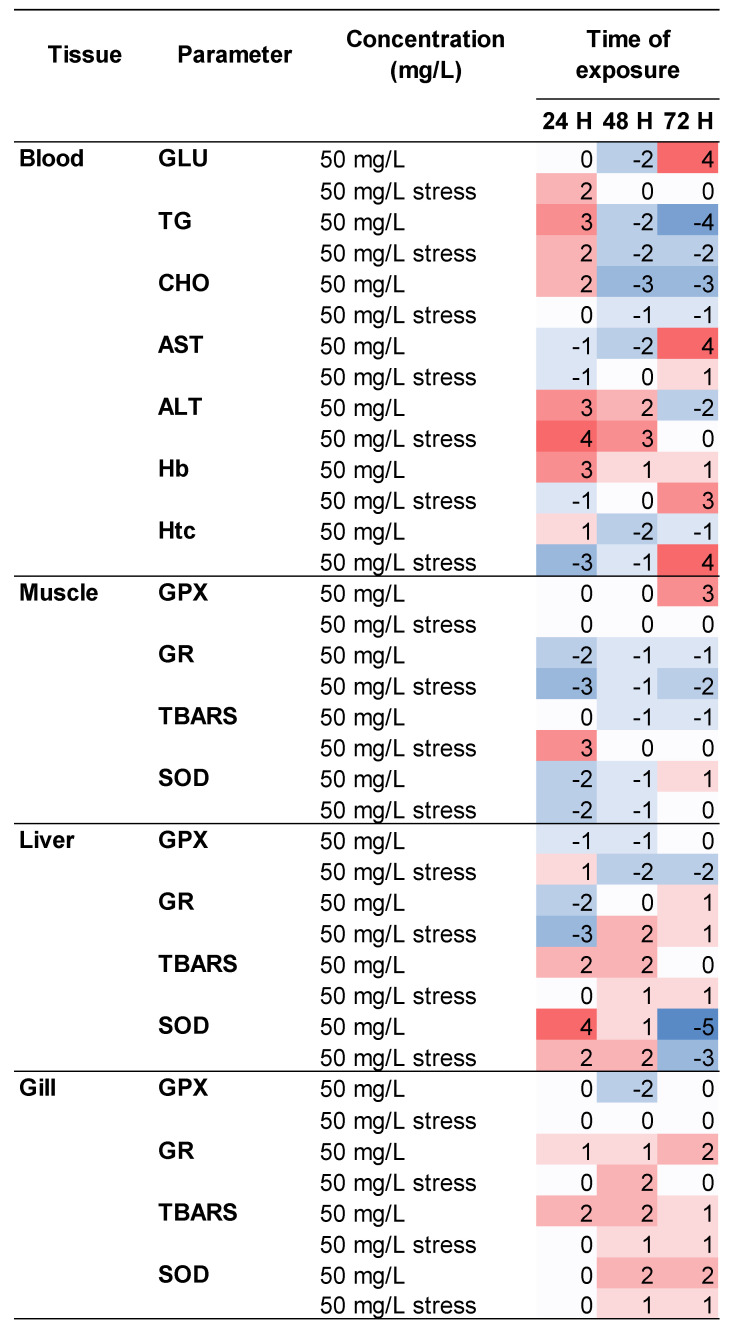
Heat map of overall modulation in mean scores over time within the various categories of the parameters analyzed in blood, muscle, liver and gill of juvenile Atlantic salmon exposed for 72 h to a microalga-derived anesthetic extract (50 mg L^−1^).

A summary diagram is presented in Figure 8. Overall stability is observed, since parameters that were more affected by exposure to anesthetic and stress at 24 h, such as GLU, TG, ALT, Htc, Hb, GR, and TBARS, at 72 h present similar levels to the CTRL group, indicating recovery or adaptation.

## 3. Discussion

It has been described that catecholamines and cortisol are released into fish blood as a primary stress response, although interspecific variation is observed in the levels recorded [1]. Secondary responses induced by these hormones lead to energy allocation to cope with the stressor; they also induce plasma hyperglycemia and an increase in other metabolites, as well as other responses. These in turn lead to increased heart rate, gill vascularization, and hydromineral imbalances. If exposure to the stressor is sustained over time, tertiary responses are observed in all systems of the organism. For example, teleosts experience metabolic disorders, reduced growth rates, immunodeficiencies, developmental alterations, reproductive disorders, or alterations in social and behavioral abilities, which clearly compromise their welfare [33,34]. Several studies have reported that anesthetics are effective in reducing stress response overall. However, there is some evidence indicating that anesthesia induces elevated levels of cortisol in some conditions [35]. Therefore, a desirable characteristic of an anesthetic is its ability to mitigate the cortisol stress response [4].

This study was performed to assess the physiological response of Atlantic salmon to prolonged exposure to a new sustainable and natural anesthetic derived from the microalga *H. akashiwo*. It was observed that exposure to the extract dosage of 50 mg L^−1^ for up to 72 h produced no negative effects in the various salmon tissues examined, indicating that the overall effect of the anesthetic extract is innocuous. Moreover, according to our results, the extract does not induce an increase in cortisol level in fish exposed to it. Similar results have been reported with other natural products used as anesthetic agents, such as nanoencapsulated clove oil in marine and freshwater species. In Atlantic salmon (*Salmo salar*), exposure to this anesthetic decreased the plasma levels of glucose and cortisol [7]. Similarly, extract of spurge (*Euphorbia rigida*), a species of flowering plant, exhibited an anesthetic effect in rainbow trout (*Oncorhynchus mykiss*) without altering plasma cortisol levels [36]. However, not all natural extracts have this characteristic. For example, in *Dicentrarchus labrax,* a seawater fish, exposure to *Eucalyptus* sp. oils could be deleterious for welfare, because they lead to an increase in cortisol level and over-expression of stress-related genes, compared with anesthetic derived from oregano oils [37]. Plasma glucose, triglycerides, and total cholesterol reflect to some extent the energy available for fast use by fish during threat response [34]. Although available cholesterol is preferably recruited by cell membranes to provide stability, in some cases (e.g., under infection) it might be mobilized to support physiological response to threats, as has been reported in the case of a relationship between high plasma cholesterol and improved disease resistance [38]. Our results showed no effect of the anesthetic extract on these parameters, suggesting that fish do not require extra energy that can be quickly mobilized for stress. The steady hemoglobin concentration and hematocrit observed in fish exposed to the anesthetic are indicative of unaltered oxygen transport capacity. These results are consistent with the evidence that after 72 h of continuous exposure there was no sign of liver damage; this was indicated by steady plasma levels of ALT and AST, markers that are normally contained in hepatocytes and are released into blood when hepatocytes are damaged. Interestingly, and in contrast to our results, other anesthetics evaluated in rainbow trout, both natural (clove oil) and synthetic (2-phenoxyethanol), induced an increase in AST activity when compared with the control group [39].

Histologically, we observed that gills were not altered by exposure either to the anesthetic or to stress. Under administration by immersion, the gills and skin are the first tissues to enter in contact with the anesthetic and represent the main route of entry and excretion of anesthetics. Therefore, as is well known, they play an essential function in the hydromineral homeostasis of fish. Fish under stressful situations may suffer alterations of the gills, such as epithelial lifting, aneurysms, increased number of goblet cells, or fusion of some secondary lamellae, causing a decrease in gill efficiency [40]. This results in a lack of oxygen supply [41] in the nearshore period. Individuals of *Salmo salar* treated with AQUI-S presented epithelial lifting from the lamellae capillary irrespective of whether they had a single or repeated dose history [42]. The results of our experiment agreed with this finding, as lamellar hyperplasia and lamellar spongiosis were the only alterations observed in gill tissues in both sedated and non-sedated fish. These findings were therefore unrelated to exposure to the anesthetic extract and were considered as part of the initial condition of fish. It may be noted here that the fish presented no clinical signs of disease, and their overall condition was considered good, based on the mandatory evaluations of the Chilean regulatory authority.

It has been reported that the anesthetics themselves can affect fish antioxidant defense systems [15]. After a simulated transportation operation, Teles et al. [43] evaluated the effects of clove oil and tricaine methanesulfonate (MS222), two of the most widely used anesthetics, on several oxidative stress-related parameters in gilthead sea bream (*Sparus aurata*). The results indicated that the use of both these anesthetic agents interferes with fish antioxidant status. In contrast, the activity of enzymes responsive to oxidative stress, such as superoxide dismutase, glutathione peroxidase and glutathione reductase, was not significantly altered in our study by continuous exposure to the anesthetic, even when fish were exposed to handling stress. There were likewise no differences in the thiobarbituric acid-reactive substances (TBARS) levels in any tissue. TBARS are used as indicator for monitoring overall lipid peroxidation, which is a helpful biochemical indicator of oxidative damage [15]. TBARS levels in tissue were significantly increased in rainbow trout exposed to the two anesthetics most commonly used in aquaculture [40]. Our positive result for oxidative stress is therefore of great importance for aquaculture, considering that the oxidative state is linked to increased susceptibility to environmental changes and different types of pathology [44].

The experimental design implemented in this study allowed us to assess the potential use of the anesthetic tested during fish transportation, a common process in the industrial rearing of salmonids [45]. Transportation of live fish is inherently stressful and entails significant risks for animal welfare. Great attention to fish welfare during transport is therefore essential, not only to ensure their survival but also to avoid increased incidence of disease resulting from weakening of the immune function and/or increased exposure to infection [46]. The stressors associated with live transportation are well documented [47]. So far, the main object has been to maintain water quality during transport to reduce stress [44]. Currently, the principal measures used by freshwater farmers include the use of anesthetics, the addition of pure O_2_, reducing the water temperature, and the addition of probiotics to functional feeds as a prophylactic measure. However, the anesthetics evaluated for use while transporting freshwater fish, namely MS222, 2-phenoxyethanol, and eugenol, were all reported to induce side effects, depending on the species [48]. In contrast, the natural anesthetic derived from *H. akashiwo* evaluated here presents negligible effects on fish and is a reliable option worth considering.

Although the toxicity of this natural anesthetic has been addressed [31], there is still more information that is under investigation to further understand the chemical composition of the product. This is relevant for the assessment of residuals in fish fillet. Here, we have searched for the *H. akashiwo* characteristic analytical fingerprint in the muscle of fish exposed to the extract and found no accumulation (preliminary data not shown), indicating that the anesthetic is most probably quickly excreted and will not represent a threat for consumers. Moreover, to demonstrate the full safety of the anesthetic, in addition to the parameters evaluated in the present study, a battery of genotoxicity indicators is under assessment. The goal is to evaluate the possible induction of genetic damage in exposed individuals [49], or in end consumers [50,51]. A comparison between a number of synthetic (e.g., benzocaine) and natural (e.g., eugenol) anesthetics showed that both might induce genotoxic effects in freshwater fish species [52]. Furthermore, it has also been demonstrated that for essential oils extracted from plants, the effect is dose-dependent. Thus, an evaluation of doses to avoid genotoxicity is needed in order to reach the market [53]. For instance, *Lippia alba* essential oil was considered safe for fish and end consumers after the assessment of its genotoxicity [50,51].

The induction of reactive oxygen species (ROS) by exposure to *H*. *akashiwo* bloom has been considered as the proximal cause of fish mortalities. In the current anesthetic study, there were no differences in the activity of antioxidant enzymes in gills of fish exposed to the extract. This was expected, since ROS are extremely unstable and they would be rapidly reduced in the medium.

As a conclusion, the anesthetic extract tested, in concentrations of 50 mg L^−1^, can be used as an alternative anesthetic for procedures with juvenile salmon. It provides induction and recovery times that fall within the established criteria for anesthetic efficiency; moreover the anesthetic is natural and does not induce side effects such as stress or metabolic damage (as shown by the blood, functional, physiological, and histological parameters measured in this study). This anesthetic is a viable option that will support fish welfare during deleterious events. Two new challenges need to be addressed in order to develop a competitive commercial product: in the first stage, the algal bioprocess need to be optimized on a larger scale to achieve industrial volumes; and in a subsequent stage, the commercial formulation needs to be evaluated at an appropriate scale for aquaculture procedures with different species.

## 4. Materials and Methods

### 4.1. Microalgal Culture and Extract Processing

The algal strain CCMP302 (New Zealand) of the species *Heterosigma akashiwo* was obtained from the Microalgae Culture Collection COPAS SUR-Austral (CCM-UdeC, Chile). It was stored and maintained at the FICOLAB center (University of Concepción, Concepción, Chile) and used for stock cultures. Algal biomass was grown in Erlenmeyer flasks (1 L) containing 500 mL of L1 medium prepared with natural filter-sterilized seawater at 33.5 PSU [54]. A batch of 10,000 cells mL^−1^ was initially inoculated from acclimated, exponential-phase stock cultures and incubated in a culture chamber at 15 ± 2 °C, with 50 µmol photon m^−2^ s^−1^ and a 16:8 (L:D) photoperiod, without aeration. Cultures were shaken manually once a day and cell density was assessed by cell counting in 1 mL Utermöhl chambers. For the purposes of biomass production up-scaling, cell batches were inoculated up to 100 L Kalwall tanks where the same growth conditions described above were maintained, adding aeration.

Algal biomass of 157,000 cells mL^−1^ at stationary growth phase, when toxicity was highest, was harvested 13 days after inoculation by adding sodium hydroxide (3M; 2.5 mL L^−1^) for high pH-induced flocculation (pH 9.4–9.6). Biomass pellets were distributed to 50 mL centrifugal tubes and centrifuged for 10 min at 5000 rpm (Eppendorf^®^5804R, Hamburg, Germany) in seawater. After removal of the supernatant, the remaining pellets were sonicated for 10 min in absolute methanol, at 47 kHz ± 6% (Branson 1210, Danbury, CT, USA) and centrifuged for 10 min at 5000 rpm. The supernatant finally recovered was the final crude extract, which presented a dark green color. The extract pH was adjusted to neutral and the volume was reduced to 1/3 parts using a rotary evaporator (IKA^®^, Staufen, Germany) at 40 °C. The concentrated extract was subjected to strong cation exchange (SCX) by loading to a solid-phase extraction (SPE) column (Strata^®^ 70 g/150 mL, 55 µm 70 Å (Boulder, CO, USA); Phenomenex^®^, Torrance, CA, USA) activated with methanol, followed by washing in ultrapure water at a load volume of 1:24 *v*/*v*. The clean extract volume was collected using a Visiprep™ SPE vacuum manifold 12 (Supelco^®^ Merck©, Darmstadt, Germany). Finally, the dried biomass was collected, weighed and resuspended in solvent (DMSO) at a concentration as low as 1.5%.

### 4.2. Experimental Design and Sampling

The in vivo experimental trial took place at the facilities of the marine biology experimental station of Dichato, Faculty of Natural and Oceanographic Sciences, University of Concepción, Chile. A group (N_Total_ = 90) of juvenile salmon smolts (*Salmo salar*), with mean total weight 132.6 g ± 7.3 g and mean total length 25.7 cm ± 4.1 cm was acclimated for 3 weeks in optimal conditions of salinity (33–34 PSU), DO (saturation 97–99%), pH (7.3–7.6), and temperature (12–14 °C), and natural photoperiod. The fish were previously screened in an independent laboratory for the presence of prevalent bacterial pathogens (e.g., *Piscirickettsia salmonis*, *Tenacibaculm* sp.) and viruses (e.g., ISAV, VHS) following the mandatory procedures of the Chilean authorities (SERNAPESCA). The procedures carried out during the trial were approved by the Bioethics Committee of the Universidad de Concepción.

**Figure 9:**
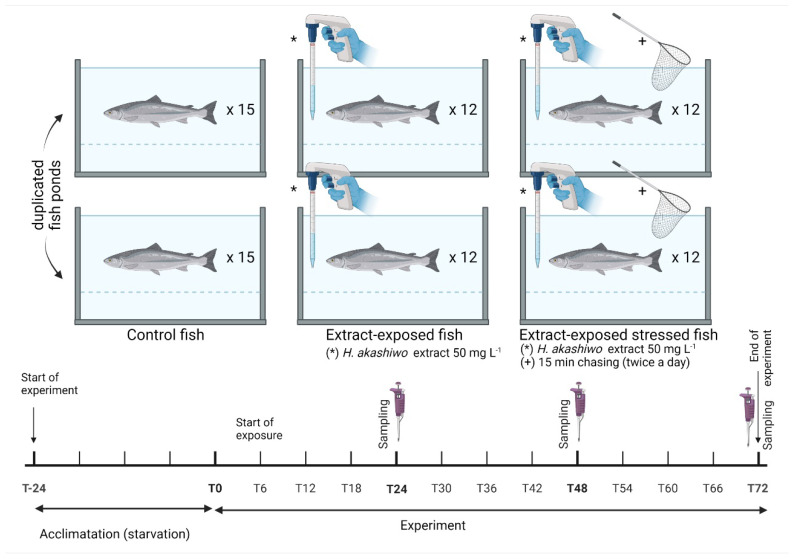
Experimental design of the in vivo trial for assessment of physiological and stress effects in juvenile salmon (*Salmo salar*) exposed for a prolonged period to a microalgal (*Heterosigma akashiwo*) extract (50 mg L^−1^) with anesthetic properties. Asterisk (*) indicates applied extract, and sign plus (+) indicates applied stress.

The fish were starved for 24 h and then distributed randomly between six tanks. Each tank was randomly assigned one of the following experimental groups: (a) control group with no exposure to algal extract or disturbance (CTRL); (b) group exposed continuously to algal extract (EXT50); and (c) group exposed continuously to algal extract and to physical handling stress (chasing with a net for 15 min) twice a day (EXT50-S). Treatments were performed in duplicate tanks and fish density was 40 kg m^−3^ in all groups. Fish were exposed to 50 mg L^−1^ of algal extract (Figure 9) and the trial was performed over 72 h in recirculation conditions. This dosage was chosen based on a preliminary trial in which salmonids were exposed for 72 h to a range of the anesthetic extract between 30 mg L^−1^ to 60 mg L^−1^ and the time to sedation was assessed. The dosage of 50 mg L^−1^ was found to be the most efficient, based on the persistence of sedative effects over time (loss of reactivity to external stimuli except to strong pressure, slight decrease in opercular rate but with normal equilibrium) and the time to recovery of regular behavior (preliminary data not shown). Sampling in all groups took place before exposure to algal extract (T0), and after 24, 48, and 72 h of exposure (T24, T48, and T72 respectively; Figure 9).

At each sampling moment, fish were quickly caught with a soft net and anesthetized with benzocaine (BZ20 20%, Europharma, Puerto Montt, Chile) following the manufacturer’s instructions. Despite the already sedated state of the fish, the use of an accepted anesthetic for sampling is a requirement of the Bioethics Committee of the Universidad de Concepción, to reduce possible suffering of animals during the process. The anesthetic chosen was one that is accepted for bioethical purposes and is regularly used in industrial aquaculture processes. Blood was collected from the caudal vein using syringes previously coated with EDTA (0.1%), and whole blood was divided into one aliquot for hemoglobin concentration analysis and hematocrit determination, and another for plasma collection by centrifuging at 5000× *g* for 15 min (MiniSpin^®^ Eppendorf^®^, Hamburg, Germany) with subsequent storage at −20 °C until processing. Fish were dissected and samples of liver, gill and muscle were quickly collected and placed in autoclaved ice-cold PBS (phosphate buffer saline, pH 7.3) and stored at −80 °C until enzymatic analysis. In addition, samples of liver, gill, anterior and posterior intestine, and skin were collected for histopathological analysis and placed in neutral buffered formalin (10% in PBS), at room temperature until processing.

### 4.3. Blood Hematology and Biochemistry Analysis

Whole blood hemoglobin was evaluated following the cyanmethemoglobin method, using the commercial kit Hemoglobin Liquicolor (Human Diagnostics, Wiesbaden, Germany) with modifications. Briefly, 5 µL of total blood were mixed with 1 mL of working reagent (0.6 mM potassium hexacyanoferrate (III), 0.7 mM potassium cyanide in 23 mM potassium bicarbonate buffer), and absorbance of the cyanmethemoglobin produced was measured at 540 nm and compared with the absorbance of standard hemoglobin processed similarly (Sigma-Merck, Darmstadt, Germany). Hematocrit was evaluated as the packed cell volume obtained after centrifugation of microhematocrit tubes at 5000 rpm for 15 min.

Plasma cortisol concentration was assessed by a solid phase competitive enzyme-linked immunosorbent assay (ELISA) using a commercial kit (EIAHCOR, Invitrogen, USA) following the manufacturer’s instructions. Analysis was performed by assessing absorbance at 450 nm and comparing it with a series of dilutions of a cortisol standard (stock at 32,000 pg mL^−1^). Plasma glucose, triglycerides, total cholesterol concentrations, as well as plasma alanine and aspartate transaminase (EC 2.6.1.2 and EC 2.6.1.1 respectively) activities were assessed using commercial kits (Human Diagnostic, Wiesbaden, Germany) following the manufacturer’s instructions. Plasma glucose was assessed by the GOD-PAP method using the Glucose Liquicolor kit; the plasma triglycerides concentration was assessed by the GPO-PAP method with lipid clearing factor Triglycerides liquicolor mono, while plasma cholesterol was assessed by the CHOD-PAP method using a Cholesterol Liquicolor kit. Alanine and aspartate transaminase activities were assessed by a kinetic method without pyridoxalphosphtate activation using the GPT (ALAT) IFCC mod. liquiUV and the GOT (ASAT) IFCC mod. liquiUV kits respectively. All kits were previously validated for their use with *Salmo salar*.

### 4.4. Enzyme Activities in Tissues

Liver, gill, and muscle from each specimen were thawed and 100 mg were dissected in an ice-cold environment and placed in a 2 mL tube with 1 mL of ice-cold 100 mM Phosphate buffer (pH 7.4). The tissues were then sonicated in ice until complete homogenization. The mixture was centrifuged at 10,000× *g* and 4 °C, and the supernatant was stored at −20 °C until analysis. The specific activities of superoxide dismutase, glutathione reductase, glutathione peroxidase, as well as lipid peroxidation in liver, gill and muscle, were assessed to evaluate oxidative stress throughout exposure to the algal extract, using commercial kits (#706002, #703202, #703102, and #700870 respectively, Cayman Chemical, Ann Arbor, MI, USA). Activities were normalized with tissue extract total proteins measured using a modified Lowry Protein Assay kit (Pierce, Thermofisher, Waltham, MA, USA) following the manufacturer’s instructions. All spectrophotometric analyses were performed using a Synergy H1 spectrophotometer (BioTek, Winooski, VT, USA).

### 4.5. Histological Analysis

In order to infer any damaging effect of the prolonged exposure to the anesthetic, histological alterations in the gills, liver and intestines were assessed by VeHiCe (Veterinary Histopathology Centre, Puerto Montt, Chile). Samples from fish exposed to the anesthetic extract for 72 h were processed using the classical histology processing pipeline, and the histopathological findings were categorized based on a semi-quantitative assessment with three levels, in which scores of 1, 2, and 3 correspond to slight, medium, and severe histopathological damage of the tissues respectively.

### 4.6. Data Analysis

Data were screened for outliers and tested for homogeneity of variance and normal distribution with the Levene’s and Shapiro Wilks tests respectively. Variables (except histological scores) were BoxCox transformed and analyzed by Two-Way ANOVA, followed by multiple comparison post-hoc Tukey’s HSD test when ANOVA was significant. Histological scores of different groups were compared with the Kruskal–Wallis non-parametric test. Significant *p*-value was set as <0.05 and all tests were performed with XLSTAT 2018 (Addinsoft, New York, NY, USA).

### 4.7. Summary Analysis by Scoring

To produce a global interpretation of the effects of the new anesthetic derived from *H. akashiwo* as a sedative/anesthetic strategy for Atlantic salmon, we adapted the results obtained for the health status and physiological response of fish into a scoring system. This system allowed comparison of the modulation dynamics of a group of parameters between the treatment and control groups. The variation from the mean of each parameter at a certain time and treatment is evaluated in relation to the mean of the same parameter in the control group. The range between the minimum and maximum mean obtained for each variable was divided into 5 equal levels in order to categorize the number of levels by which a group’s score exceeded or fell short of the control, with scores ranging between −5 and +5. In other words, if a group had a score of −3 (for example), this indicates that the mean value of the variables was 3 levels lower than in control group, while a score of 0 indicates that mean value was similar to that of the control.

## Data Availability

Not applicable.
